# Antioxidant and Gastroprotective Activity of *Suaeda fruticosa* Forssk. Ex J.F.Gmel

**DOI:** 10.3390/molecules27144368

**Published:** 2022-07-07

**Authors:** Afsheen Ayaz, QurratUlAin Jamil, Musaddique Hussain, Fayyaz Anjum, Adeel Sarfraz, Taha Alqahtani, Nadia Hussain, Reem M. Gahtani, Ayed A. Dera, Hanan M. Alharbi, Shahid M. Iqbal

**Affiliations:** 1 Department of Pharmacology, Faculty of Pharmacy, The Islamia University of Bahawalpur, Bahawalpur 63100, Pakistan; afsheenayaz31@gmail.com (A.A.); musaddique.hussain@iub.edu.pk (M.H.); fayyaz.anjum@hotmail.com (F.A.); 2 Department of Pharmacy Practice, Faculty of Pharmacy, The Islamia University of Bahawalpur, Bahawalpur 63100, Pakistan; quratulain.jamil@iub.edu.pk; 3 Department of Anatomy and Histology, Faculty of Veterinary and Animal Sciences, The Islamia University of Bahawalpur, Bahawalpur 63100, Pakistan; adeel.sarfraz@iub.edu.pk; 4 Department of Pharmacology, College of Pharmacy, King Khalid University, Abha 62529, Saudi Arabia; ttaha@kku.edu.sa; 5 Department of Pharmaceutical Sciences, College of Pharmacy, Al Ain University, Al Ain 64141, United Arab Emirates; nadia.hussain@aau.ac.ae; 6 Department of Clinical Laboratory Sciences, College of Applied Medical Sciences, King Khalid University, Abha 61421, Saudi Arabia; rmalqahtani@kku.edu.sa (R.M.G.); ayedd@kku.edu.sa (A.A.D.); 7 Department of Pharmaceutics, College of Pharmacy, Umm A-Qura University, Makkah 21955, Saudi Arabia; hmsharbi@uqu.edu.sa

**Keywords:** gastroprotective, antioxidant, *Suaeda fruticosa*, mucin contents

## Abstract

*Suaeda fruticosa* Forssk. Ex J.F.Gmel is traditionally used for inflammatory and digestive disorders, as a carminative, and for diarrhea. This plant is widely distributed in Asia, Africa, and the Mediterranean region. Aqueous methanolic extract of *S. fruticosa* (Sf.Cr) was prepared and screened for phytoconstituents through qualitative and GC-MS analysis. Quantification of total phenolic and flavonoid contents was performed, while antioxidant capacity was determined by DPPH, CUPRAC, FRAP, and ABTS assays. The gastroprotective activity was assessed in an ethanol-induced ulcer model. Gastric secretory parameters and macroscopic ulcerated lesions were analyzed and scored for ulcer severity. After scoring, histopathology was performed, and gastric mucus contents were determined. Oral pre-treatment of Sf.Cr demonstrated significant gastroprotection. The gastric ulcer severity score and ulcer index were reduced while the %-inhibition of ulcer was increased dose-dependently. The Sf.Cr significantly elevated the pH of gastric juice, while a decrease in total acidity and gastric juice volume was observed. Histopathology demonstrated less oedema and neutrophil infiltration in gastric mucosa of rats pre-treated with the Sf.Cr in comparison to ethanol-intoxicated animals. Furthermore, the gastric mucus contents were increased as determined by alcian blue binding. Sf.Cr showed marked gastroprotective activity, which can be attributed to antioxidant, antisecretory, and cytoprotective effects.

## 1. Introduction

A gastric ulcer is an erosion in the mucosal lining of the stomach affecting 5–10% of the global population. This number increases dramatically to 50% at the age of 60 years [1]. It develops when the injurious mechanisms surpass the protective mechanisms of gastric mucosa—i.e., mucus and bicarbonate secretion. The injurious mechanisms include factors that either erode the mucosal layer directly or facilitate the erosion. Endogenous factors include acid, pepsin, surfactants, refluxed bile salts, and weakened mucosal defense, while exogenous factors include drugs like NSAIDs, pathogenic organisms such as *H. pylori*, cigarette smoking, and alcohol consumption [2]. Ulcer prevalence due to heavy alcohol consumption is 7.6% in the USA [3]. Multiple pathways are involved in damage to the gastric mucosa by alcohol exposure [4]. Major ulcer complications include bleeding, perforation, penetration, and gastric outlet obstruction. A hemorrhage is caused when ulcer erosions extend to an artery or vein. Perforation is a life-threatening complication that is caused when the ulcer aggravates, resulting in severe damage to the mucosal wall that allows luminal contents to leak into the abdominal cavity [5,6]. In some cases, the gastric ulcer penetrates to the pancreas, lesser omentum, liver, biliary tract, colon and mesocolon [7]. Gastric outlet obstruction can occur due to the mechanical impediment of gastric emptying [8].

Pharmacological therapy for gastric ulcers includes acid suppression by H^+^/K^+^ pump inhibitors, H_2_ antagonists, and cytoprotection by drugs that act by strengthening the mucosal barrier [9]. Since a gastric ulcer is a condition caused by suppressed defensive mechanisms, the therapy is targeted at increasing the gastric mucosal protection against injurious agents. Regardless of the fact that drugs that suppress acid secretion have been the mainstay of therapy, the high cost and adverse effects of prolonged treatment as well as ulcer recurrence have resulted in the need to search for new therapeutic agents that could protect against ulcers while having fewer adverse effects [10]. Recurrence of gastric ulcers occurs frequently, and the recurrence rate after 10 years of therapy is as high as two-thirds of the total cases [11].

Medicinal plants have been described as inhibiting gastric ulcers through various mechanisms in animal models. They can be seen as a valuable source for new drug development. *Suaeda fruticosa* Forssk. Ex J.F.Gmel is a perennial succulent plant that belongs to the family Amaranthaceae, which falls under the genera *Suaeda*. *S. fruticosa* is a widely distributed plant in Pakistan, India, Iran, Afghanistan, the Arabian Peninsula, the Atlantic coasts of southern Spain and Portugal, the Mediterranean region, France, south-eastern England, and in the peninsula of north-east Africa [12]. In Pakistan, it is locally known as Laani or Khaar and its flower, fruit, leaves, and seeds are taken orally either as a powder, decoction, juice, or mixed with the oil. The plant has been traditionally used for gastrointestinal disorders including diarrhea and as a carminative. It is also used as an antibacterial, for rheumatism, gout, conjunctivitis, flu, cough, and skin diseases [13,14]. The plant exhibits antioxidant [15], anti-inflammatory [16], hepatoprotective [17], hypolipidemic, hypoglycemic [18], and anti-cancer activities [14]. The *Suaeda* group of plants like *S. monoica* and *S. heterophylla* have been traditionally used in ulcer therapy [19,20]. *S. fruticosa* has also been used in gastrointestinal disorders; however, no gastroprotective activity has been reported. In the context of this background, we evaluated the antioxidant and potentially gastroprotective effects of *S. fruticosa*.

## 2. Results

### 2.1. Extraction and Phytochemical Analysis of Sf.Cr

The extraction was carried out by using one kilogram of powdered plant material. After solvent removal, 98.8 g of Sf.Cr plant extract was obtained, which gave approximately 9.88% yield. The phytochemical assay of Sf.Cr indicated the presence of saponins, terpenes, phenols, tannins, carbohydrates, and flavonoids.

### 2.2. GC-MS Analysis

**Figure 1:**
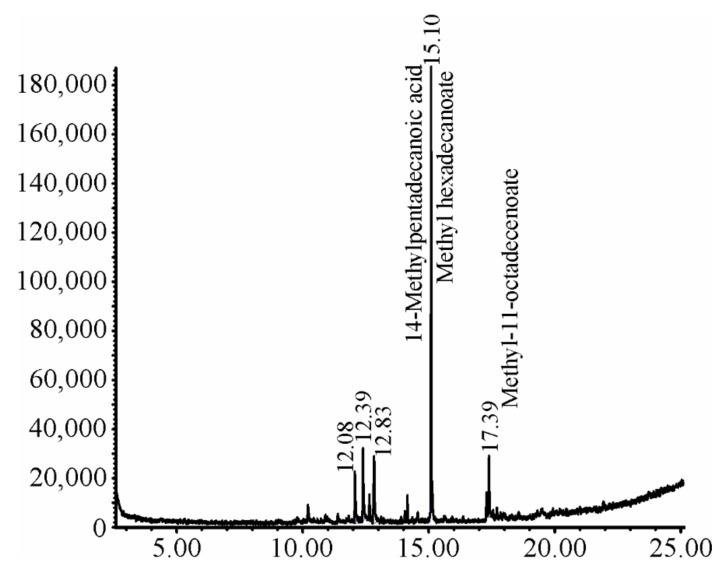
GC-MS chromatogram of Sf.Cr. The sample was injected at 220 °C having the scanning range of 70–700 *m*/*z* and compounds were identified by comparing retention time and mass fragmentation using NIST 2014 mass spectral library.

Three compounds were identified by GS-MS analysis of Sf.Cr. These compounds were 14-methylpentadecanoic acid (95%), methyl hexadecenoate (93%), and methyl-11-octadecenoate (86%). The total area of the first two compounds was 34.90%, while for the last compound it was 65.10% (Figure 1).

### 2.3. Antioxidant Activity

#### 2.3.1. Total Phenolic and Flavonoid Contents

The phenolic contents of Sf.Cr were determined by the Folin–Ciocalteu assay by using gallic acid as a reference. The TPC determined were 205 ± 23.52 mg GAEg^−1^ dry weight (DW) of the Sf.Cr. The flavonoid contents of Sf.Cr were determined by an aluminum chloride assay and quercetin was used as a reference. The TFC determined were 90.02 ± 6.88 mg QEg^−1^ DW of the Sf.Cr.

#### 2.3.2. Reducing Potential Assay

The reducing potential of Sf.Cr was determined by CUPRAC and FRAP assays. Results are expressed as mg equivalent of trolox per gram of Sf.Cr (mg TEg^−1^). Sf.Cr showed CUPRAC activity of 562.23 ± 89.98 mg TEg^−1^ Sf.Cr, while in the FRAP assay it was 234.12 ± 3.03 mg TEg^−1^ Sf.Cr.

#### 2.3.3. Radical Scavenging Assay

Sf.Cr was subjected to DPPH and ABTS assays. The results of these assays are expressed as milligram equivalents of trolox per gram of Sf.Cr (mg TEg^−1^). Sf.Cr showed DPPH-based free radical scavenging activity of 124.71 ± 12.44 mg TEg^−1^ dry extract. The ABTS assay displayed radical scavenging potential of 40.84 ± 1.24 mg TEg^−1^ Sf.Cr.

### 2.4. Gastro-Protective Effect of Sf.Cr against Acute Ulcer

#### 2.4.1. Effect on Gross Evaluation of the Gastric Mucosa

The gastroprotective effects of Sf.Cr were evaluated in an ethanol-induced ulcer model. Animals were placed into six different experimental groups—i.e., normal control, intoxicated, standard, and 30, 100, and 300 mg/kg Sf.Cr treatment groups.

**Figure 2:**
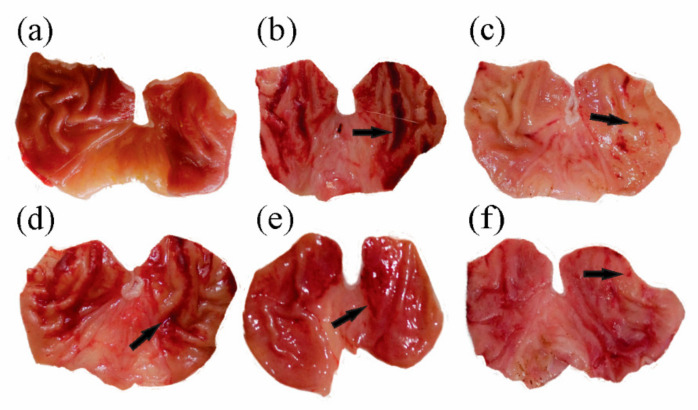
Representative images showing ethanol-induced gastric ulceration in rat’s stomach where arrowheads indicate macroscopic lesions and petechiae. (**a**) Normal control group showing normal morphology, (**b**) intoxicated group showing widespread hemorrhagic lesions, (**c**) sucralfate (100 mg/kg) pre-treatment group showing few lesions and spot ulcers, (**d**) 30 mg/kg, (**e**) 100 mg/kg, and (**f**) 300 mg/kg Sf.Cr pre-treatment group showing dose-dependent gastroprotection.

There were no visible lesions, and gastric mucosa appeared normal in the control group (Figure 2a). The intoxicated group (Figure 2b) showed extensive gastric mucosal damage characterized by macroscopic lesions on the gastric wall and numerous petechiae, mostly confined in the gastric corpus. The standard group received sucralfate (100 mg/kg), which prevented the formation of lesions, and only small petechiaes were present (Figure 2c). Pre-treatment with the Sf.Cr exhibited a dose-dependent reduction in the mucosal damage. The dose of 30 mg/kg Sf.Cr showed widespread hemorrhagic lesions having gastric mucosal injuries comparable to that of the intoxicated group (Figure 2d). The 100 mg/kg Sf.Cr treatment reduced the length of lesions as well as the thickness of the hemorrhagic streaks (Figure 2e). The 300 mg/kg treatment displayed few lesions which were comparable to the sucralfate treatment group (Figure 2f). The mucosal folds of the stomach were also flattened in the 300 mg/kg treatment.

**Figure 3:**
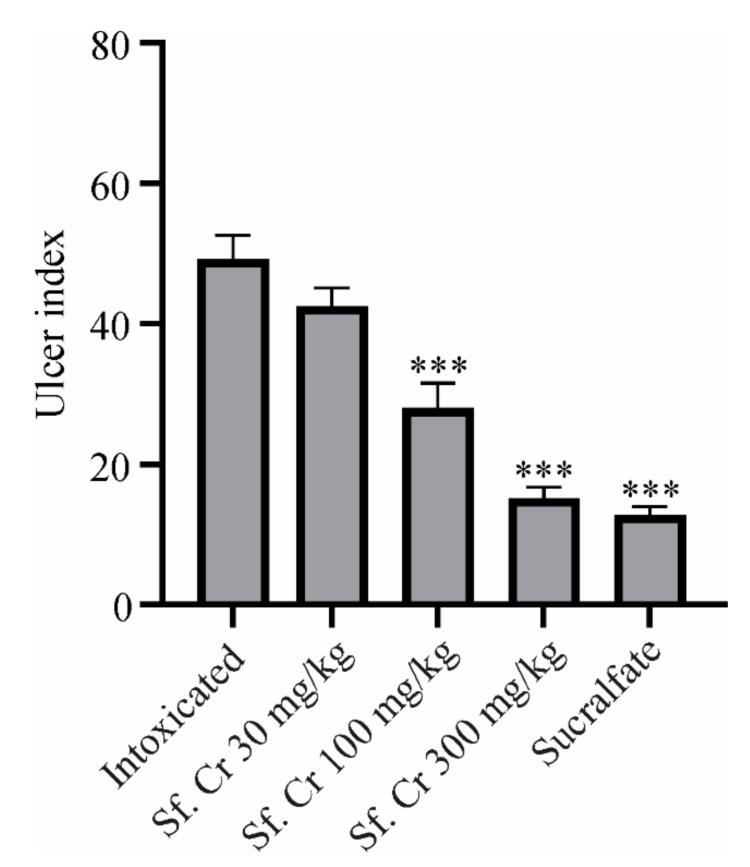
Effect of Sf.Cr on ulcer index. Rats were pre-treated orally with normal saline, sucralfate, and Sf.Cr. Data are presented as Mean ± SEM (n = 6). Significance was determined by One way ANOVA followed by Dunnett’s test and described as (***) if *p* < 0.001 compared with the intoxicated animals.

To assess the damage quantitatively, gastric lesions were scored (Table 1) from the gross morphology of the stomach for the determination of the ulcer severity index using the Guth scoring standard [21]. The ulcer index (UI) indicates the degree of damage caused by the necrotizing agents. The treatment with sucralfate significantly (*p* value < 0.001) reduced the UI value when compared to the intoxicated group. The Sf.Cr treatment groups showed dose-dependent reductions in UI. The groups treated with 100 and 300 mg/kg showed significantly (*p* value < 0.001) reduced UI compared to the intoxicated group (Figure 3).

The UI was also expressed as a percentage of protection against gastric ulcers after ethanol administration. The treatment with sucralfate displayed the highest protection of 73.90%, while the Sf.Cr treatment groups observed a dose-dependent effect. The maximum gastroprotective effect was observed with 300 mg/kg—i.e., 67.54%, which was comparable to the sucralfate group. The difference between the two groups was statistically non-significant. Pre-treatment with 100 and 30 mg/kg showed 43.57 and 11.19% protection, respectively. The UI values and gastroprotective effects were consistent with those of gross examination, which displayed that the rats in the intoxicated group developed ulcers as evidenced by the gross morphology, including lesions, congestion, and hemorrhagic petechiaes, while the rats that received sucralfate or Sf.Cr 100 and 300 mg/kg exhibited a reduction in the lesions of the gastric mucosa.

#### 2.4.2. Effect on Gastric Secretory Parameters

Gastric contents were collected from the dissected stomachs in the graded centrifuge tubes to determine the volume, pH, and total acidity of gastric juice. The ethanol administration caused an increase in gastric secretions with gastric volume of 7.3 ± 0.17 mL. The pre-treatment with sucralfate and Sf.Cr significantly reduced gastric secretions (Table 1) when compared with the intoxicated group. The pH of gastric secretion was increased in the sucralfate and Sf.Cr treatment groups (Table 1). The total acidity was calculated and showed the highest value of 40.58 ± 1.23 mEq/L/100g in the intoxicated group. Pre-treatment with sucralfate showed the lowest total acidity, while Sf.Cr reduced the total acidity of gastric juice in a dose-dependent manner. A significant decrease in total acidity was observed in 100 and 300 mg/kg Sf.Cr treatment groups compared to the intoxicated group (Table 1).

#### 2.4.3. Effect on Gastric Mucus Contents

**Figure 4:**
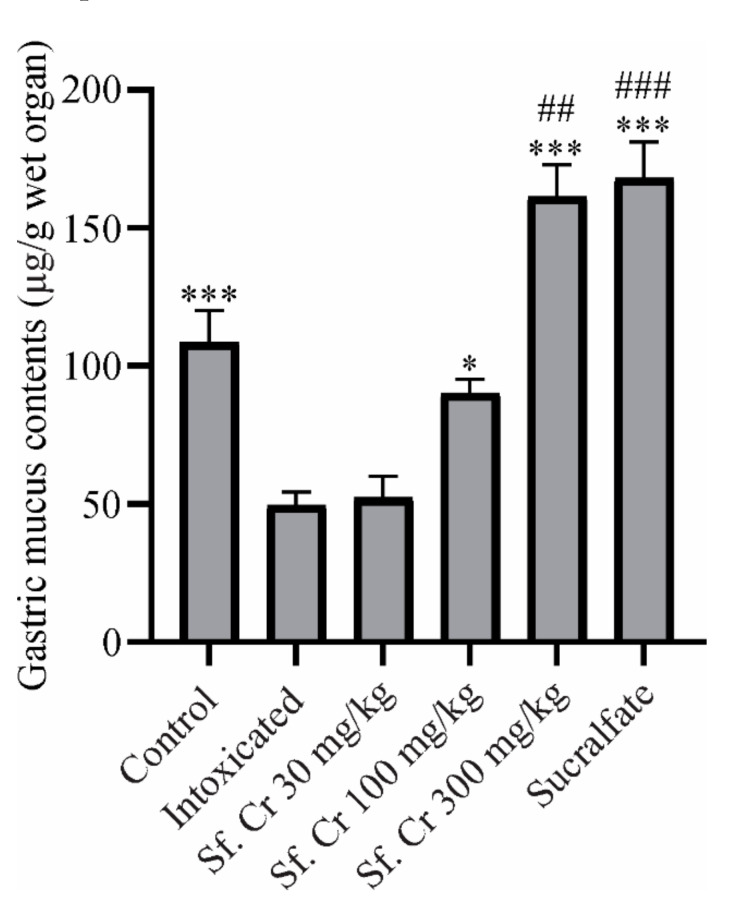
Effect of Sf.Cr on gastric mucus contents. Values are expressed as Mean ± SEM. Significance was determined by one way ANOVA followed by Dunnett’s test. Values are considered as significant (*) if *p* < 0.05, highly significant (***) if *p* < 0.001 compared with intoxicated group, and (##) if *p* < 0.01, (###) *p* < 0.001 compared with normal control group.

The adherent gastric mucus contents were determined by Alcian blue staining. Oral administration of ethanol decreased the mucus contents while the sucralfate protected the gastric mucosa from the necrotizing effects of ethanol by increasing the mucus contents. Pretreatment with 100 and 300 mg/kg Sf.Cr also increased gastric mucus contents, thereby resulting in gastroprotection (Figure 4). This increase in mucus content was significant compared to both the normal control and intoxicated groups.

### 2.5. Histopathological Assessment of Gastric Damage

**Figure 5:**
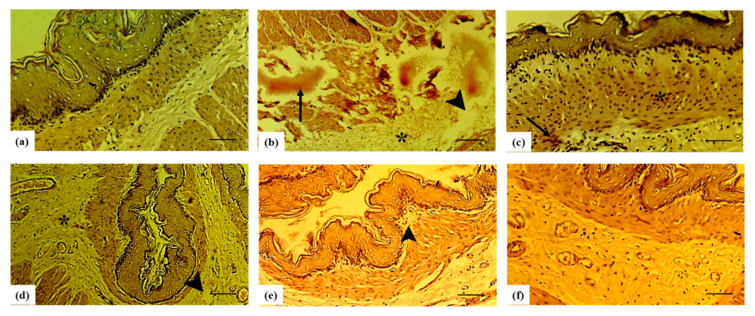
Histomicrographs of the rat stomach with H & E stain at magnification of 400X (scale bar: 100 µm). Control group (**a**) shows normal morphology and no visible signs of inflammation; intoxicated group (**b**) showing several inflammatory changes including bloody spots (arrow), oedema (arrowhead), and polymorphic nuclear cell infiltration (*). The stomach mucosa and submucosa showed fewer sings of inflammation when treated with sucralfate however infiltration and bloody spots are visible (**c**) and Sf.Cr showed amelioration of stomach tunics in a dose dependent manner with inconspicuous inflammatory changes (**d**–**f**).

The normal histomorphology of rat stomachs had the typical mucosa having crypts, mucus-secreting cells of gastric glands with rounded nuclei. The lamina propria was intact, with infiltrated lymphocytes that were fewer and were scattered uniformly, along with normal vasculature and fibrous connective tissue. Collagen fiber content was within normal histological limits (Figure 5a). The histomicrographs of the stomach showed an altered profile with visible signs of inflammation in the ulcer-induced group. The stomachs showed visible inflammation, with bloody spots and ulceration. The ulcer formation was deep in the gastric mucosa and extended to the basement membrane. The polymorphous lymphocytes and fibrin were also noted. The hyperplastic gastric glands surrounded the ulcer. There were higher numbers of lymphocytes and polymorphonuclear leucocytes in lamina propria (Figure 5b). Upon treatment with sucralfate, ulcerations in the stomach were notably reduced and even a normal morphological picture was noted with very few signs of inflammation and degenerative changes (Figure 5c). Sf.Cr pre-treatment ameliorated the mucosa and wearing of superficial erosions, recovering the basement membrane. Fewer inflammatory cells were found in the lamina propria. Sf.Cr pre-treatment also minimized the necrosed mucosa, submucosal oedema, and hemorrhage. The hyperplasia of the gastric glands was also decreased (Figure 5 d–f). The healing was dose dependent; however, there were no significant differences in the histological profile of Sf.Cr 100 and 300 mg/kg pre-treatment (Table 2).

## 3. Discussion

Gastric ulcer is a common treatable clinical situation which affects patient quality of life and causes economic burdens on the health care system. It occurs due to weakened defensive mechanisms in gastric mucosa. Changing lifestyles, including smoking, alcohol consumption, over the counter use of NSAIDs, and *H. pylori* infection have increased the prevalence of gastric ulcers. Alcoholics have 1.43 times the risk of upper gastrointestinal bleeding compared to non-alcoholics, and this risk is further potentiated with the concurrent use of NSAIDs. Alcohol causes congestion, hemorrhagic lesions with microvascular damage, oedema, and exfoliation of the epithelium [22,23], which may be due to the overproduction of ROS [24], reduced prostaglandins, and release of inflammatory mediators [25]. Several antioxidant-rich substances have demonstrated gastroprotective effects against ethanol-induced gastric injury [26,27]. Medicinal plants possess a number of biological activities such as anti-oxidant, anti-inflammatory, anti-secretory, and cytoprotective, which collectively protect and heal gastric ulcers through prophylactic and therapeutic mechanisms [28]. The present study describes the gastroprotective effects of *S. fruticosa* Forssk. Ex J.F.Gmel, a medicinal plant from the family Amaranthaceae. Traditionally, this plant has been used to treat gastrointestinal disorders and exhibits antioxidant and anti-inflammatory activities [13,29].

A preliminary screening of Sf.Cr indicated the presence of different active phytoconstituents including tannins, saponins, flavonoids, phenols, terpenes, and carbohydrates. GC-MS analysis of Sf.Cr showed the presence of 14-methylpentadecanoic acid, methyl hexadecenoate, and methyl-11-octadecenoate. We were unable to identify other lipophilic compounds because we analyzed crude aqueous methanolic extracts, which could be considered a limitation of this study. Ideally, fractionation by solvents with increasing polarity followed by GC-MS analysis of non-polar fractions will improve identification [30]. Previously, UHPLC-MS analysis of methanolic extracts of *S. fruticosa* indicated the presence of 11 different secondary metabolites from different classes, including octadecanoic acid, which we also observed in our GC-MS analysis [31]. Similarly, another research group performed LC-MS analysis of a 70% aqueous-ethanolic extract of *S. vermiculata* and found nine different compounds, including flavonoids and one fatty acid—hexadecanoic acid—which we also observed in our GC-MS analysis [32]. These branched chain fatty acids have also been identified in other plants exhibiting gastroprotective activity [33], and they are also described for their anti-inflammatory activity [34].

The gastroprotective potential of Sf.Cr was determined in an ethanol-induced ulcer model and was compared against sucralfate. The protective effect was evaluated on the basis of the macroscopic structure, which demonstrated a reduction in grossly visible gastric mucosal lesions (Figure 2). Deep necrotic lesions were either reduced or absent in Sf.Cr pre-treated rats at doses of 100 and 300 mg/kg. Lesions observed were thinner and smaller in 100 mg/kg Sf.Cr pre-treated rats compared to the intoxicated rats. There were small spot ulcers, and flattening of gastric mucosal folds was observed in 300 mg/kg Sf.Cr pre-treated rats. This flattening of gastric rugae could be due to reduced gastric motility. Previous studies have shown that reduced gastric motility contributes to ulcer protection. Distension of circular muscles results in a flattening of rugal folds, thereby leading to a wider gastric mucosal area and thus decreasing the volume of ulcerogens on rugal folds [35]. Similarly, in another study, it was ascertained that flattening of rugal folds in ethanol ulcerated gastric tissue played a role in prevention of ulcers [36]. An ulcer severity score was assigned by determining the lesion length and width (Table 1). Intoxicated rats showed the highest ulcer score, as the hemorrhagic lesions produced were thicker and larger. However, a dose-dependent reduction in severity of ulcer lesions was observed in Sf.Cr treated rats. The pre-treatment with 300 mg/kg Sf.Cr produced results comparable to the standard drug sucralfate. A decrease in the UI is directly related to a reduction in the severity of gastric ulcers. Our study reported that ethanol intoxication produced a pronounced increase in the ulcer index (UI), while pre-treatment with sucralfate and Sf.Cr caused a significant reduction in the UI in a dose-dependent manner. The pre-treatment group that was given 100 mg/kg Sf.Cr exhibited a UI of 28, while the 300 mg/kg Sf.Cr pre-treatment group exhibited a UI of 15.17, which is comparable to the 12.83 UI of sucralfate pre-treatment (Figure 3). These results were also supported by the histopathology of the gastric tissue (Figure 5; Table 2).

The oral pre-treatment of Sf.Cr produced a marked reduction in gastric juice volume, whereas intoxicated rats exhibited an increase in the level of gastric juice. Compared to the ulcer-induced group, pre-administration of sucralfate and Sf.Cr doses of 100 and 300 mg/kg protected gastric mucosa by significantly elevating the pH of gastric juice (Table 1). It is noteworthy that when the pH level reached 3.5, the UI reduced significantly. Sf.Cr could exert gastroprotective action by increasing alkalinity through mucus production and bicarbonate secretion. Research data showed that flavonoids play a role in increasing the gastroprotection by decreasing acid release and elevating the pH levels [37,38]. Gastric acidity is responsible for the physiological function of the digestive system. However, a huge increase in the acidity of gastric juice can contribute to ulcer development. Ethanol intoxication increased the total acidity (Table 1). A mild decrease in total acidity was observed with 30 mg/kg, while 100 and 300 mg/kg showed a pronounced decrease. Intoxication by ethanol aggravated the acid secretion, thereby resulting in increased gastric volume and acidity, decreased gastric pH, and increased lesions, as depicted by the increased UI. Sf.Cr reduced the acid secretion, which resulted in decreased volume as well as total acidity and an increase in the pH of the gastric juice, and it also decreased the ulcer lesions as demonstrated by a decrease in the UI. This shows the involvement of the antisecretory mechanisms in the protective activity of Sf.Cr against ethanol-induced ulcers.

In gastric ulcers, despite low acid secretion, weakened protective mechanisms can lead to mucosal injuries. Gastric mucosa is continuously exposed to physical and chemical noxious agents including gastric acid, pepsin, certain foods, microbial toxins, abrasive substances, refluxed bile, pancreatic juice, and irritating drugs. Mucus is regarded as a first line of defense against such harmful agents; it protects gastric mucosa by forming an insoluble gel-like coating over the epithelial cells and together with bicarbonate acts as a shield. It also contributes to inhibition of mechanical destruction by providing a microenvironment over exposed sites where rapid healing by regeneration of epithelial cells can occur [39]. Several drugs offer gastroprotection by stimulating mucus secretion, including sucralfate [40], carbenoxolone [41], misoprostol [42], and bismuth compounds [43]. Several plants have also been reported to display gastroprotection by increasing mucus contents, thus indicating that the antiulcerogenic activity of the plants is linked to the strengthening of the mucosal barrier [33]. Damage to the gastric tissue is accompanied by a decrease in adherent mucus contents. Pre-treatment with Sf.Cr 100 mg/kg increased the mucus contents, though they were not significantly different from the normal control group. However, a dose of Sf.Cr 300 mg/kg significantly increased the mucus contents that were comparable to the sucralfate group. Increased mucus levels are responsible for the cytoprotective activity that improves gastric acid buffering, strengthens the barrier against acid back diffusion, and decreases the gastric wall traction during peristalsis and contractions [44]. It can be postulated that augmented mucus contents by Sf.Cr contributes to the gastroprotective activity.

The exposure of gastric mucosa to necrotizing agents such as absolute ethanol can bring pathological changes, thereby leading to the development of inflammation and hemorrhagic disruptions with significant involvement of free radicals. Studies have reported that antioxidants play an active role in defending the cellular damage from ROS by scavenging free radicals. Quantification of phenolic and flavonoid contents in Sf.Cr showed higher amounts of TPC and TFC. Sf.Cr possesses high free radical scavenging and reducing properties evidenced by DPPH, ABTS, CUPRAC, and FRAP assays. This antioxidant activity can be attributed to the high levels of phenols and flavonoids present in the Sf.Cr, which also protect gastric mucosa from the deleterious effects of alcohol by countering ROS.

## 4. Materials and Methods

### 4.1. Collection of Plant and Extract Preparation

The *S. fruticosa* plant was collected in the month of November from Cholistan desert (29°22′31.4″ N, 71°46′24.9″ E), Pakistan. The botanical identity of the plant was determined by a botanist. A specimen of the collected plant was assigned a voucher number of SF-WP-08-21-196 and was submitted for further reference to the herbarium of Pharmacology Research Laboratory, Faculty of Pharmacy, the Islamia University of Bahawalpur, Pakistan.

The crude extract of *S. fruticosa* was prepared as described previously [45]. Briefly, the plant was cleaned from dust, rinsed, dried, and ground to obtain coarse powder. One kg powdered material was extracted using 70% methanol; solvent was evaporated by a rotary evaporator (Heidolph, laborata 4000-efficient, Schwabach, Germany,) at 35–40 °C. Complete drying of the extract was carried out in a hot air oven (Memmert GmbH, Schwabach, Germany) at 40 °C. The crude extract of *S. fruticosa* (Sf.Cr), was labeled, weighed, and its percentage yield was calculated. Sf.Cr was stored in a freezer at −20 °C.

### 4.2. Phytochemical Analysis and In-Vitro Antioxidant Activity

Qualitative analysis of various phytoconstituents such as flavonoids, alkaloids, quinones, amino acids, glycosides, steroids, phenols, saponins, tannins, and carbohydrates was performed as described previously [46].

Total phenolic contents (TPC) and total flavonoid contents (TFC) of Sf.Cr were quantified by the Folin–Ciocalteu phenol assay and aluminum chloride assay [45,47]. Briefly, 1 mL of Sf.Cr (1 mg/mL) was mixed with 1mL of the diluted Folin–Ciocalteau’s phenol reagent and allowed to stand for 5 min in the dark. After 5 min, 10 mL of sodium carbonate (7%) was added to this solution. Then, 13 mL of distilled water was added and mixed thoroughly. The solution was incubated in the dark for 60 min, and absorbance was taken at 750 nm. Gallic acid in different concentrations was used to draw the calibration line. For TFC, an aliquot of Sf.Cr 0.3 mL (300 µg/mL in methanol) was mixed with 0.15 mL of 0.5 M NaNO_2_, 0.15 mL 0.3 M AlCl_3_.6H_2_O, and 3.4 mL of methanol (50%). After 5 min, 1 mL of 1 M NaOH was added. The solution was mixed thoroughly, and absorbance was measured at 506 nm. The same procedure was repeated for different concentrations of quercetin, and the calibration line was constructed to calculate total flavonoid contents.

The radical scavenging activity of Sf.Cr was determined by ABTS and DPPH assays, while CUPRAC and FRAP assays were performed to assess the reducing power of Sf.Cr as described previously [48]. Briefly, the DPPH assay was performed by mixing 1.0 mL of Sf.Cr (1 mg/mL) with 4 mL of DPPH (0.267 mM), and then placed in the dark for 30 min at room temperature, followed by the measurement of absorbance at 517 nm. The ABTS assay was performed by a reaction mixture of ABTS (7 mM) and potassium persulfate (2.45 mM). Methanol was used to dilute this solution until absorbance of 0.70 ± 0.02 at 734 nm was obtained. Then, 1 mL of Sf.Cr (1 mg/mL) and 2 mL of the working ABTS^+^ solution was mixed and kept for 30 min, and absorbance was measured at 734 nm. For the CUPRAC assay, cupric chloride (1 mL, 10 mM), buffered by 1 mL ammonium acetate buffer at pH 7.0 (1 M), were mixed with neocuproine (1 mL, 7.5 mM). In this reaction mixture, 0.5 mL of Sf.Cr (1 mg/mL) was added and placed at room temperature for 30 min, after which absorbance was measured at 450 nm. For the FRAP assay, a 0.3 M acetate buffer (pH 3.6), 10 mM TPTZ, and 20 mM ferric chloride (10:1:1 ratio) were mixed with 40 mM HCl. About 0.1 mL of Sf.Cr (1 mg/mL) and 0.9 mL of the FRAP working solution was kept at room temperature for 30 min, followed by absorbance measurement at 593 nm using a UV-1800, Shimadzu, Japan, UV-Visible spectrophotometer. Results are expressed as milligram equivalent of trolox per gram of Sf.Cr (mg TEg^−1^).

### 4.3. GC-MS Analysis

Analysis of the extract was performed on the GC-MS system (Agilent Technologies, Santa Clara, CA, USA) with an auto-sampler by preparing the sample as described previously [49]. Experimental conditions were as follows: HP-5MS ((5%-phenyl)-methylpolysiloxane) capillary standard non-polar column; dimension length: 30 m, internal diameter: 0.25 mm; outfitted with an Agilent HP-5973 mass selective detector (Ionization energy: 70 eV). The 2 µL of sample was injected in the GC column with a flow rate of helium maintained at 0.8 mL/min. The temperature was initially set at 80 °C, and then raised at a rate of 10 °C/min to 280 °C. The temperature of the injector was 220 °C, while the scanning range was 70–700 *m*/*z*. The compounds were identified by comparison of relative retention time and mass fragmentation using NIST 2014 mass spectral library [49].

### 4.4. Experimental Animals

Albino wistar rats weighing 200–250 g of either sex were housed in a bioterium. Standard conditions were maintained, with a temperature of 22 ± 1 °C and relative humidity of 45–55% along with a 12 h light/darkness cycle. Water and standard diet were freely accessible to the animals. Rats were kept for 15 days under the described environmental conditions in the laboratory for acclimatization prior to the study to curtail stress. The procedures involving animal studies were approved (PAEC/21/50) by the Pharmacy Animals Ethics Committee of Faculty of Pharmacy, The Islamia University of Bahawalpur.

### 4.5. Induction of Gastric Ulcer

Absolute ethanol (5 mL/kg) was administered to induce acute gastric ulcers as previously described with minor modifications to evaluate the gastroprotective effects of Sf.Cr [50]. Animals were randomly placed into six groups, with six animals in each group. The grouping was as follows: Group-I: normal control was given normal saline 10 mL/kg; Group-II: intoxicated; Groups III, IV, and V were treated with Sf.Cr in increasing doses (30, 100 and 300 mg/kg). The standard group—viz., Group VI—received sucralfate 100 mg/kg body weight of animal. Groups II–VI received absolute ethanol for ulcer induction 1 h post-treatment. After one hour of ulcer induction, rats were anesthetized by ketamine and xylazine (50:5 mg/kg) for 5 min and euthanized by cervical dislocation. Stomachs were isolated from each rat. Gastric contents were collected in graduated centrifugation tubes and subjected to centrifugation for 10 min at 1000 rpm. The volume of supernatant was noted, and the pH was measured with the help of a pH meter (InoLab pH 720). Titration was carried out by using 0.01 N sodium hydroxide. The volume of sodium hydroxide was noted to calculate the total acidity (mEq/L/100g) [51].


Total acidity=(volume of NaOH used×normality of NaOH)/0.1×100

### 4.6. Determination of Gastric Ulcer Severity

The stomachs were excised, cut open along the greater curvature, and carefully washed using normal saline to remove blood clots or cellular debris to observe macroscopic lesions. The flattened stomach samples were laid on a clean white sheet and photographed using a digital camera. The length and width of the ulcerated lesions were measured using software for image analysis (ImageJ, NIH, Bethesda, MD, USA). Ulcers were scored for severity of gastric lesions using the Guth scoring standard [21]. The ulcer index was calculated by dividing the total scores of each group with the number of animals. The ulcer score is the number given to each group for total epithelial lesions considering the length and width of the lesion.

The percentage protection was determined using the following equation:


%protection=((ulcer index)control−(ulcer index)test)/((ulcer index)control)×100

### 4.7. Gastric Mucus Contents

Mucus contents were determined by the previously described method with minor modifications [52,53]. The dissected stomach was cut along the greater curvature, washed, and divided into two equal segments. One section was dipped in 10% formalin for histopathological examination. The glandular part of the other section of the stomach was cut and weighed. It was soaked in a 0.1% Alcian blue solution prepared in 0.25 M sucrose and 0.05 M sodium acetate (pH 5.8) buffer. The Alcian blue solution was freshly prepared and filtered before use. To remove the non-complexed dye, the glandular part was washed twice for 15 and 30 min with a 0.25 M sucrose solution. The complexed dye was eluted by immersing the stomach portion in a 0.5 M magnesium chloride solution with occasional shaking. Then, this solution was extracted by an equal amount of diethyl ether by vigorously shaking for 2 min until emulsion formation. The emulsion was centrifuged for 10 min, and absorbance of the aqueous layer was measured at 580 nm. A standard curve of Alcian blue (6.25–100 µg/mL) was drawn, and results were presented as µg of Alcian blue/g wet organ.

### 4.8. Histopathology

For histopathological examination, formalin fixed stomach tissue was embedded in paraffin blocks for sectioning using microtome. Staining of the obtained sections (4 µm) was performed with standard hematoxylin and eosin (H&E) stain. These slides were anonymously evaluated and scored by a histopathologist.

### 4.9. Statistical Analysis

The results were described as Mean ± SEM, and significance was calculated by using one-way ANOVA followed by post hoc Dunnett’s test where appropriate. *p* < 0.05 was set as the significance level. A statistical software GraphPad Prism was used to analyze the data.

## 5. Conclusions

To conclude, the current study explored the gastroprotective properties of *S. fruticosa* in an acute ulcer model. Sf.Cr pretreatment reduced the grossly visible gastric mucosal lesions. It displayed a dose-dependent decrease in the ulcer severity score and ulcer index (UI) and increased the % protection. The Sf.Cr showed antisecretory activity demonstrated by increased pH, reduced total acidity, and gastric juice volume. An increase in the adhered mucus contents of rat gastric tissue indicates that Sf.Cr exerted cytoprotective effects. Histopathological studies demonstrated significant improvement in gastric tissue. Sf.Cr also possesses good antioxidant activity, which contributes in gastroprotection. However, additional extensive studies are required to isolate and chemically characterize the plant in order to describe the phytochemicals responsible for gastroprotection.

## Figures and Tables

**Table 1:** Effect of Sf.Cr and sucralfate on ulcer score, pH, gastric volume, and total acidity in ethanol-induced gastric ulcer.

Treatment	Ulcer Score	pH	Gastric Juice Volume (mL)	Total Acidity (mEq/L/100g)
Normal control	00	4.03 ± 0.04	1.7 ± 0.07	21.50 ± 1.16
Intoxicated	295	1.94 ± 0.05	7.3 ± 0.17	40.58 ± 1.23
Sf.Cr 30 mg/kg	255	2.13 ± 0.03 *	6.5 ± 0.32 *	37.50 ±0.76 *
Sf.Cr 100 mg/kg	168	3.50 ± 0.04 ***	3.5 ± 0.16 ***	24.13 ± 0.32 ***
Sf.Cr 300 mg/kg	91	4.21 ± 0.07 ***	2.1 ± 0.05 ***	18.02 ± 0.42 ***
Sucralfate	77	4.50 ± 0.02 ***	1.6 ± 0.02 ***	13.50 ± 0.45 ***

Rats pre-treated with normal saline 10 mL/kg (normal control), Sf.Cr at dose of 30, 100 and 300 mg/kg, sucralfate (100 mg/kg) one hour before ulcer induction. Mean ± SEM (*n* = 6) is used to show the data. Significance was determined by One way ANOVA followed by Dunnett’s test. Values are considered as significant (*) if *p* < 0.05, and highly significant (***) if *p* < 0.001 compared to the intoxicated group.

**Table 2:** Effect of Sf.Cr and sucralfate on histomorphology of ethanol-induced gastric ulcer tissue.

Histopathological Lesions	Normal Control	Intoxicated	Sucralfate 100 mg/kg	Sf.Cr 30 mg/kg	Sf.Cr 100 mg/kg	Sf.Cr 300 mg/kg
Necrosis of gastric mucosa	00	03	02	02	02	02
Mucosal inflammatory cells infiltration	00	02	01	02	01	02
Submucosal inflammatory cells infiltration	00	02	02	02	02	02
Submucosal oedema	00	02	02	01	02	01
Hemorrhage	00	03	01	02	01	01
Hyperplasia of gastric glands	00	02	02	01	01	01
Degree of ulceration	00	02	01	02	01	01
Total score	00	16	11	12	10	10

Rats were pre-treated with normal saline 10 mL/kg (normal control), Sf.Cr at doses of 30, 100 and 300 mg/kg, sucralfate (100 mg/kg) one hour before ulcer induction. Rats were euthanized and stomachs were isolated and stored in formalin. Stomach tissues were sectioned and stained with H&E stain followed by anonymous evaluation and scoring by a histopathologist.

## Data Availability

Not applicable.
